# Preoperative ultrasound detection of arteria lusoria and non-recurrent laryngeal nerve anatomical variation: a retrospective study

**DOI:** 10.1055/a-2731-9908

**Published:** 2026-01-16

**Authors:** Jean-Baptiste Safa, Marc Tassart, Jean-Noel Talbot, Pierre-Yves Marcy, Sophie Périé, Isabelle Thomassin-Naggara, Alexandre Faure

**Affiliations:** 1 Radiology 55705 Hopital TenonParisÎle-de-FranceFrance; 2 Nuclear Medicine 55705 Hopital TenonParisÎle-de-FranceFrance; 3 Interventional Radiology and Radiodiagnostic DepartmentPolyClinics ELSAN MedipoleOllioulesFrance; 4 Oto-Rhino-Laryngology 532063 Groupe Hospitalier Privé Ambroise Paré-HartmannNeuilly-sur-SeineÎle-de-FranceFrance

**Keywords:** right non-recurrent laryngeal nerve, cervical ultrasonography, cervical surgery, laryngeal paralysis, arteria lusoria

## Abstract

**Purpose:**

Arteria lusoria (AL), a rare vascular variant (prevalence of 0.5–3.2%), is linked to a right non-recurrent laryngeal nerve (RNRLN), thereby increasing nerve injury risk during thyroid and parathyroid surgery. This study evaluates ultrasonography (US) for preoperative AL detection.

**Materials and Methods:**

Retrospective analysis of 1406 hyperparathyroidism patients undergoing 18F-fluorocholine PET/CT with subgroup analysis in 270 patients with “expert US” performed on-site. An experienced senior radiologist assessed the US Y-image (brachiocephalic artery division), with low-dose CT as the standard of truth for AL confirmation.

**Results:**

18F-fluorocholine PET/CT analysis found 22 cases of AL (prevalence: 1.57%). US identified 259 Y-images, excluding AL, confirmed by CT. Five absent Y-images indicated AL, all were confirmed by CT. Six non-assessable Y-images were AL-negative on CT. US performance: sensitivity 100% (95% CI: 0.463–1), specificity 98% (95% CI: 0.95–0.99), negative predictive value (NPV) 100%, accuracy 98%.

**Conclusion:**

US Y-image assessment reliably excludes AL with an NPV of 100%, offering a non-invasive tool to reduce RNRLN injury risk in thyroid and parathyroid surgery.

## Zusammenfassung

**Ziel:**

Die Arteria lusoria (AL), eine seltene Gefäßvariante (Prävalenz 0,5–3,2%), ist mit einem nicht rezidivierenden rechten Nervus laryngeus (RNRLN) assoziiert und erhöht somit das Risiko einer Nervenverletzung während einer Schilddrüsen- und Nebenschilddrüsen-Chirurgie. Diese Studie untersucht den Stellenwert der Ultraschalluntersuchung (US) zur präoperativen Detektion der AL.

**Material und Methode:**

Retrospektive Analyse von 1406 Patienten mit Hyperparathyreoidismus, die sich einer 18F-Fluorcholin-PET/CT unterzogen. Subgruppen-Analyse bei 270 Patienten mit vor Ort durchgeführtem „Experten-US“. Ein erfahrener Oberarzt für Radiologie beurteilte das US-Y-Bild (Teilung des Truncus brachiocephalicus), wobei eine Niedrigdosis-CT als Referenzstandard zur Bestätigung der AL diente.

**Ergebnisse:**

Die 18F-Fluorcholin-PET/CT-Analyse identifizierte 22 AL (Prävalenz: 1,57%). Im Ultraschall wurden 259 Y-Bilder ohne Nachweis einer AL gefunden, bestätigt durch CT. Fünf fehlende Y-Bilder wiesen auf eine AL hin, die alle durch CT bestätigt wurden. Sechs nicht beurteilbare Y-Bilder waren in der CT AL-negativ. Die US-Leistung: Sensitivität 100% (95%-KI: 0,463–1), Spezifität 98% (95%-KI: 0,95–0,99), negativer prädiktiver Wert (NPV) 100%, Genauigkeit 98%.

**Schlussfolgerung:**

Die Beurteilung des US-Y-Bildes schließt das Vorliegen einer AL zuverlässig mit einem NPV von 100% aus – und stellt somit ein nicht invasives Verfahren dar, um das Risiko einer RNRLN-Verletzung während einer Schilddrüsen- und Nebenschilddrüsen-Chirurgie zu reduzieren.

## 1. Introduction

Vocal fold paralysis due to recurrent laryngeal nerve (RLN) injury remains the main complication of thyroid and parathyroid surgery 1 . The RLN is a mixed nerve that arises from the vagus nerve. On the right side the vagus nerve becomes the right RLN (RRLN) after looping under the right subclavian artery (RSCA). The RRLN ascends on the right side of the trachea up to the first tracheal ring ( Fig. 1 a), before innervating the vocal cord muscles, allowing phonation. On the right side, the embryological development of the RLN is intertwined with the brachiocephalic artery (BCA), which commonly originates from the aortic arch before splitting into the RSCA and the right common carotid artery (RCCA). The Y-shaped common division of the BCA is described in medical imaging as a Y-image or “Y-sign” 2 .

**Fig. 1:**
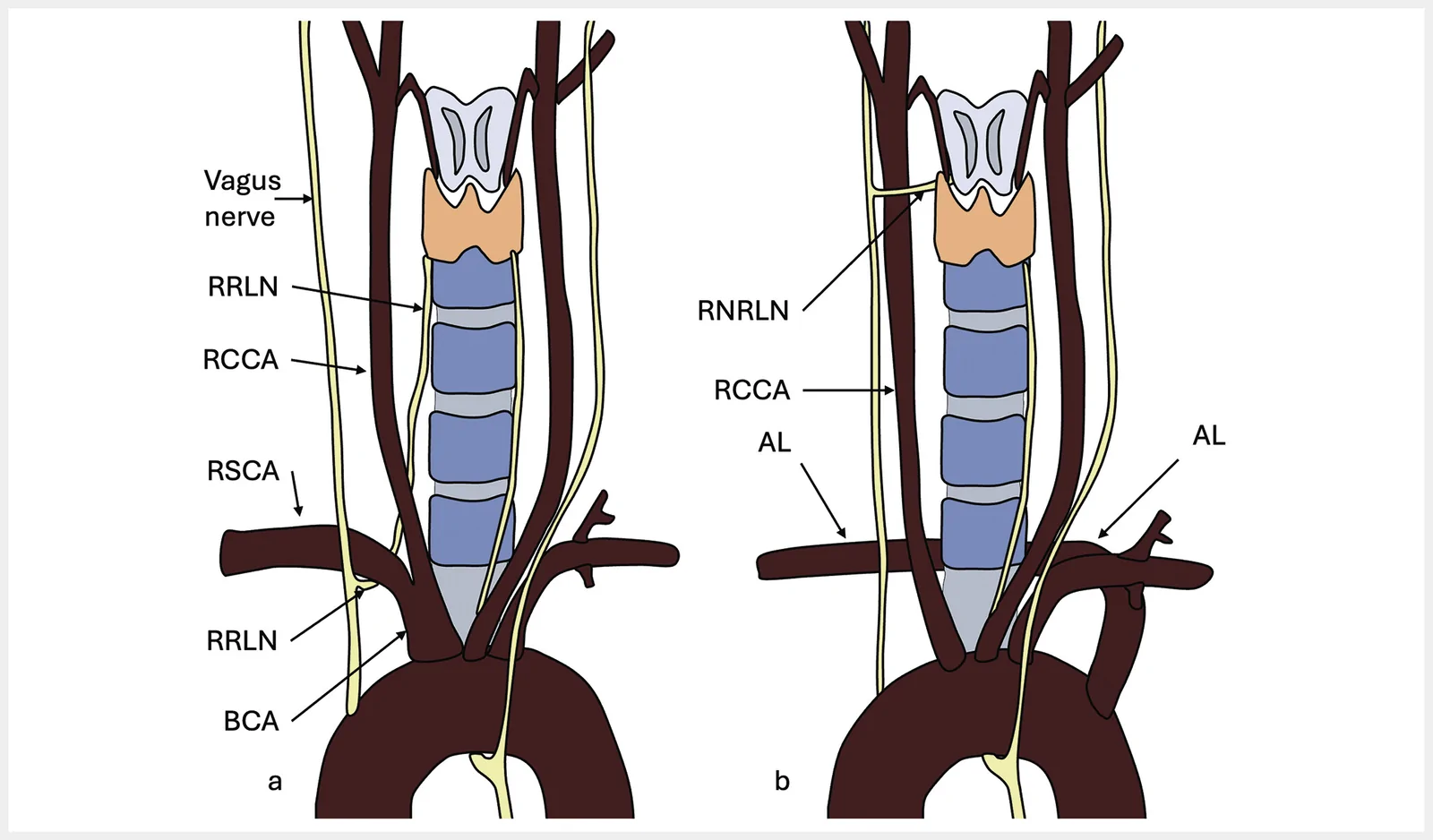
Anatomy. **a** ) Normal BCA’s division with vagus nerve becoming the RRLN with a vertical ascending trajectory by trachea’s right side. **b** ) AL arising directly from the aortic arch and the RNRLN with a horizontal trajectory. AL: arteria lusoria; BCA: brachiocephalic artery; RCCA: right common carotid artery; RRLN: right recurrent laryngeal nerve; RSCA: right subclavian artery.

AL is a rare anatomical variant (prevalence of 0.5% to 3.2%) 3 4 5 , resulting from an anomaly during development of the pharyngeal arches 6 , leading to an aberrant RSCA, originating directly from the aortic arch. The AL then posteriorly crosses the esophagus before reaching the right axillary region ( Fig. 1 b). Almost all AL cases 7 are associated with a right non-recurrent laryngeal nerve (RNRLN) 5 8 . The RNRLN originates from the vagus nerve, at a higher position than the normal RRLN, with a horizontal trajectory towards the larynx.

If the surgeon is unaware of an RNRLN, risks of intraoperative nerve damage and subsequent vocal fold paralysis increase (12% vs 1.8%) 9 10 , highlighting the importance of preoperative anatomical assessment 11 12 . Preoperative AL detection would predict an existing RNRLN, allowing surgeons to adapt their surgical protocol, thereby reducing nerve damage risks and surgery duration 13 .

One prospective study explored the use of preoperative US for the specific detection of AL to predict RNRLN and found an accuracy of 100% compared to CT scan 4 . Three retrospective studies explored the preoperative US prediction of RNRLN with surgery as the standard of truth and found an accuracy between 98% and 100% and a reduction in operating time 13 14 15 .

This study focuses on the exclusion of AL using the simple identification of the Y-image on day-to-day routine systematic cervical ultrasonography to avoid any unnecessary CT scans and to help reduce surgical complications.

## 2. Materials and methods

### 2.1 Study design and PET/CT procedure

We retrospectively analyzed the preoperative imaging workup of 1406 patients with primary or secondary renal hyperparathyroidism who underwent an ^18^ F-fluorocholine (FCH) PET/CT scan performed with the same PET/CT machine according to the same protocol at our center between May 2016 and 2022 ( Fig. 2 ). 270 of them had their cervical US examination performed by a single radiologist who was an expert in otorhinolaryngology imaging.

**Fig. 2:**
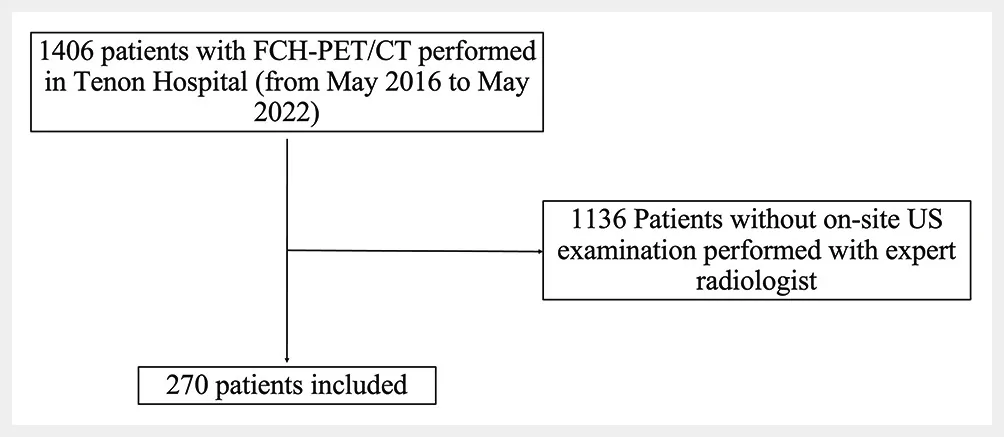
Flow chart.

Retrospective studies derived from this database without any further interaction with patients were approved by the ethics committee of our institution.

All patients benefited from an FCH-PET/CT scan using the same time of flight PET/CT machine in 3D acquisition mode (Biograph mCT 20/4R). Image acquisition was started on average 20 min after injection of 3 MBq/kg of body mass of FCH. The low-dose CT scan component with native contrast of the PET/CT examination had the following technical characteristics: 20 slices, 140kVp acquisition, use of the CARE Dose4D algorithm for adaptation of the current.

### 2.2 US procedure

All 270 “expert” cervical US examinations were performed in order to identify parathyroid glands (PT) by systematically assessing the thyroid gland. The presence or absence of the Y-image (defined as the division of the right subclavian and right common carotid arteries from the brachiocephalic artery) was mentioned in standardized on-site reports. Different sonograms from various manufacturers were used: Aloka (ProSound α10), Toshiba (APPLIO 500), GE (Voluson E8, E10, GE Logiq E10,) Canon (Aplio i800, Aplio MX).

A large multi-frequency superficial linear array (8–18 MHz) was used for superficial analysis of the thyroid and retrothyroid areas. A systematic complementary analysis was performed using either a micro-convex (6–11 MHz) or an endo-cavitary probe (7–11 MHz). The probe was positioned transversally on the cervical notch. After identification of the RCCA, high- or low-frequency arrays were angled caudally to search for the division of the BCA, i.e. the Y-image.

A standardized US report was written for each patient mentioning the presence or absence of the Y-image, based on the following criteria:

- Y-image present: identification of the RSCA and RCCA division from the BCA ( Fig. 3 a).
- Y-image absent: no identification of the RSCA and RCCA division from the BCA, suggesting AL ( Fig. 3 b).

**Fig. 3:**
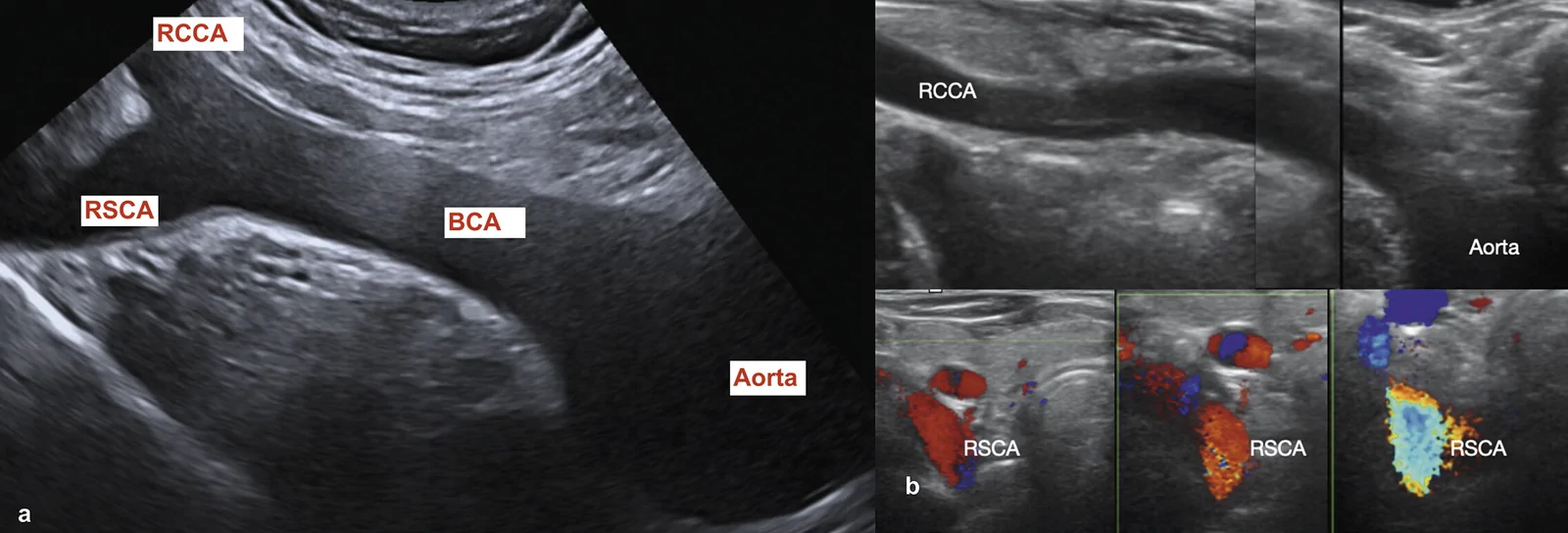
US exploration of BCA’s division. **a** ) Presence of Y-image reflecting BCA’s modal division. The probe is positioned transversally on the cervical notch. After identification of the RCCA, high or low frequency is angled caudally, searching for the division of the BCA. **b** ) Absence of Y-image with an RCCA arising directly from the aortic arch, and an RSCA with postero-anterior blood flow.

The surgeon was aware of the US report, so particular attention was given in cases of an absent Y-image.

### 2.3 CT scan as standard of truth

To determine US performance with respect to AL detection, another radiologist retrospectively analyzed all low-dose CT scan components of PET/CT examinations, without knowledge of the US results.

The standard of truth for AL was its direct visualization on the CT scan with native contrast ( Fig. 4 ).

**Fig. 4:**
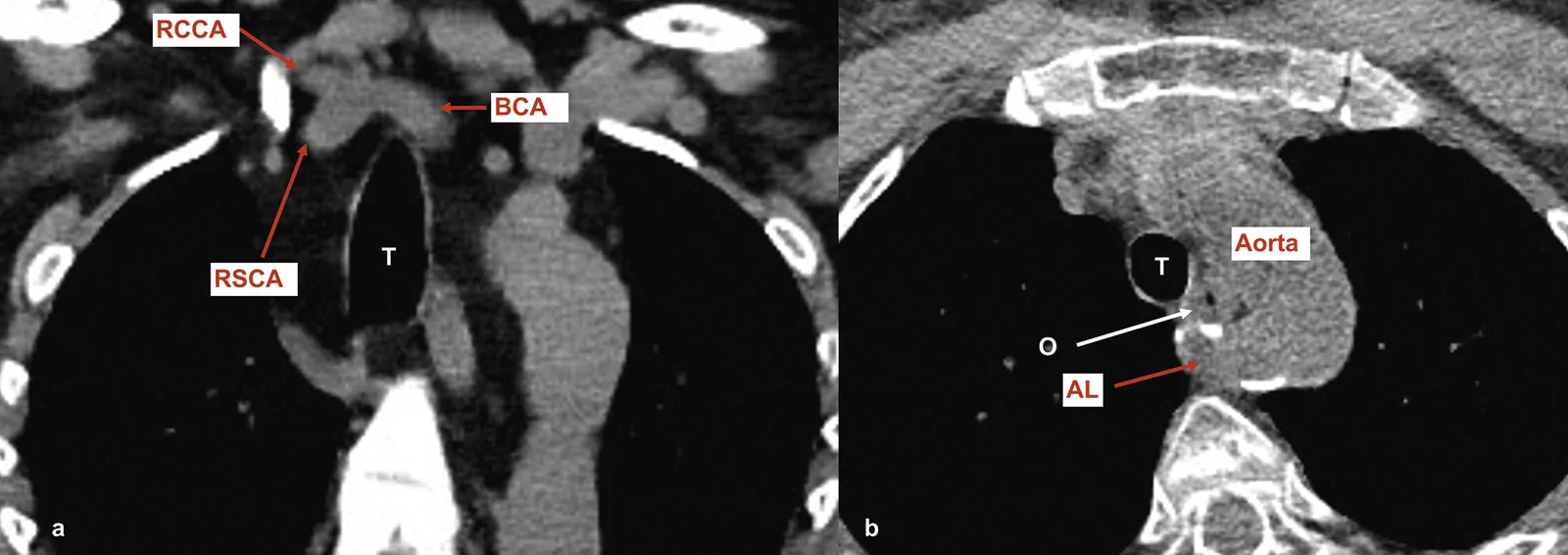
CT scan exploration of BCA’s division. **a** ) Coronal CT reconstruction with normal BCA’s division (CT Y-image). **b** ) AL = RSCA, originating directly from the aortic arch, posteriorly crossing the esophagus and trachea.

PET/CT reports did not mention the presence of AL, so the surgeon was not alerted in relevant cases.

### 2.4 Statistical analysis

Continuous variables were reported as medians and interquartile range (IQR), and categorical variables were expressed as numbers (percentages).

Performance analysis for US prediction of AL (sensitivity, specificity, positive predictive value [PPV] and negative predictive value [NPV]) with 95% confidence interval was performed.

## 3. Results

The analysis of the CT scans of the FCH PET/CT examinations in the whole series of 1406 patients revealed a total of 22 cases of AL (17 without the expert US mentioning the AL), yielding a 1.57% (95% CI: 0.91; 2.2) prevalence for AL.

The subgroup with the “expert” US cohort included 270 patients (74% female with a median age of 60 [IQR: 48–70]). There was a female-to-male ratio of 2:1, mostly due to a recognized gender bias in the case of primary hyperparathyroidism.

Retrospective analysis of hybrid FCH-PET/CT low-dose CT scan components confirmed 5 cases of AL out of 270 CT scans, yielding a 1.85% (95% CI: 0.24; 3.46) prevalence for AL in this subgroup ( Table 1 ). The prevalence of AL was similar in this subgroup and in the rest of the cohort (chi-square-test p>0.6).

**Table:** **Table 1** Principal diagnostic test’s parameters.

	Absent US Y-image	US Y-image	
Arteria lusoria on CT	5 TPs	0 FNs	Sensitivity (95% CI): 1 (0.463; 1)
Normal BCA division on CT	6 FPs	259 TNs	Specificity (95% CI): 0.98 (0.95; 0.99)
	PPV (95% CI): 0.45 (0.18; 0.75)	NPV (95% CI): 1 (0.98; 1)	Prevalence: 1.85%
TP: true-positive; FP: false-positive; TN: true-negative; FN: false-negative; PPV: positive predictive value; NPV: negative predictive value; CI: confidence interval; US: ultrasonography; BCA: brachiocephalic artery			

A US Y-image was not seen in 11 patients in total, with 5 patients having AL confirmed by CT scan (true positives) ( Table 1 ).

For the 6 remaining patients without a US Y-image who had no AL on CT (false-positives): 2 were overweight, 2 had a voluminous thyroid goiter, 1 had both, and the last one presented with a lower than usual division of the BCA in the mediastinum, behind the sternum. The trajectory of the RSCA was most likely not able to be assessed on US.

No patients with a Y-image on US had an AL on CT (false-negatives).

This resulted in the following diagnostic performance for the US prediction of AL: a sensitivity of 1 (95% CI: 0.463; 1), a specificity of 0.98 (95% CI: 0.95; 0.99), a PPV of 0.45 (95% CI: 0.18; 0.75), and an NPV of 1 (95% CI: 0.98; 1) ( Table 1 ).

Overall, the accuracy was 98%.

All 5 patients with AL detected on “expert” US underwent surgery. After review of their postoperative reports, none of the 5 patients suffered from intraoperative nerve damage or subsequent vocal fold paralysis:

- Three of them had a right abnormal PT resected (1 adenoma, 2 hyperplastic PTs) and presented higher nerve damage risks. RRLN was surgically unidentified (surgeons were aware of the very high suspicion of RNRLN) and none of the postoperative reports confirmed the existence of RNRLN through direct visualization.
- The remaining 2 cases of AL underwent surgical intervention for left PTs and were not at risk of RNRLN damage.

Of the 17 patients without expert US mentioning AL, 9 were treated surgically (right-side exploration in 8 cases), without any intraoperative nerve damage or subsequent vocal fold paralysis (RRLN was not mentioned in 5 cases and explicitly not found in 3 cases).

## 4. Discussion

Our retrospective cohort presented a female-to-male ratio of 2:1 – due to a known female predominance in primary hyperparathyroidism.

Five of the 270 patients in the expert US subgroup had an AL, yielding a prevalence of AL similar to that of the whole cohort of 1406 patients, i.e., 1.57% (95% CI: 0.91; 2.2).

All AL cases identified on CT in this subgroup were detected on US based on the absence of the Y-image, giving a sensitivity of 1 (95% CI: 0.463; 1). The corresponding PPV for an AL was 0.45 (95% CI: 0.18; 0.75). In the presence of a Y-image, US excluded the possibility of an AL with an NPV of 1 (95% CI: 0.98; 1).

None of the 5 patients with AL experienced nerve damage following surgery. The local surgeons were aware of the very high suspicion of AL after a US examination was unable to find a Y-image.

Our 1.57% prevalence for AL falls within the range of previous studies (0.5% to 3.2%) 3 4 5 16 17 .

Previous RNRLN surgical incidence findings ranging between 0.3% and 0.8% 10 14 are in contrast with higher AL prevalences of up to 3.16% observed in other studies 3 . RNRLNs are probably underestimated by surgeons unaware of AL: Some surgically unfound RRLNs are most likely RNRLNs missed by surgeons, due to their aberrant trajectory. In cases of failure to surgically identify the RRLN in its expected location, or when doing a PT surgery with PT glands so superficial that RRLN/RNRLN dissection is not required, surgeons should consider an existing RNRLN, sometimes found in different dissection planes. Two different RNRLN positions have been described 14 18 19 :

- One where the nerve arises from the cervical vagus and runs together with the superior thyroid pedicle at the upper pole of the thyroid gland.
- Another where the nerve arises at a lower level, sometimes by the thyroid isthmus 20 and runs transversely under the trunk or between the branches of the inferior thyroid artery 14 17 .

The wide 95% CI for sensitivity (0.463; 1) reflects the low number of cases of AL in the “expert US” subgroup of our cohort due to its low prevalence.

Our Y-image US detection excluded the possibility of an AL with an NPV of 1 (95% CI: 0.98; 1), which is concordant with the literature 4 8 . Our 6 false-positives, accounting for a low PPV of 0.45 (95% CI: 0.18; 0.75), were due to patients being overweight or obese, to voluminous thyroid goiters, or to a low localized division of the BCA behind the sternum, suggesting a need for complementary imaging in such cases. The literature is very divergent on this subject, with a PPV ranging from 4% 21 to 46% 15 and even 100% 4 . The probes that were used and population morphotype variations are a possible explanation to this extreme range.

Another cause for false-positives has been described in cases with an overly tortuous BCA 14 . A possible cause of false-negatives, which was not observed in our study but reported in anatomical descriptions 22 , corresponds to the right vertebral artery originating from the RCCA (false Y-image), observed in cases of AL. It has a smaller diameter than the RSCA and a higher origin, by the thyroid gland, with a vertical trajectory towards transverse processes of the vertebrae. Similarly, 30% of cases of AL have been associated with a common trunk for left and right common carotids 7 22 originating from the aortic arch, easily distinguishable from the Y-image by following the cervical trajectories of both carotids. Other extremely rare and unusual vascular variants without a right Y-image include 6 :

- Situs-inversus, where the RCCA and RSCA have separate aortic origins, and patients have a left BCA with a Y-shaped division.
- Right aortic arches where all supra-aortic arteries have separate distinct origins 23 .

In our 5 AL postoperative reports, no RNRLN was surgically confirmed, even though the surgeon was aware of the US results. No vocal fold paralysis was reported. All of our patients benefited from a minimally invasive inferior parathyroidectomy. In cases of upper positioned RNRLNs (first type described above), the RNRLN might stay out of the surgeon’s trajectory. Surgeons would have to dissect the vagus nerve and identify the RNRLN from its origin to confirm its existence.

In the study by Iacobone in 2015 15 , patients without a preoperative US examination had a significantly higher rate of surgically unidentified RRLN/RNRLN of 2.67% compared to 1.14% when a preoperative US examination was performed. Nonetheless, they had a higher prevalence of RNRLN of 1.94% for patients with a preoperative US examination compared to the prevalence of 0.67% in the control group without a preoperative US examination. The retrospective analysis of 163 preoperative CT scans conducted by Hermans et al. 3 showed an AL prevalence of 3.16% (5 cases), although AL was never mentioned in the initial reports. Only one RNRLN was surgically confirmed and one of these patients suffered from immediate and permanent unilateral vocal fold paralysis.

Preoperative imaging assessment is different before PT vs. thyroid surgery:

- Our patients suffering from HPT benefited from a recommended combination of US and a functional imaging modality 24 , allowing comparison between US and CT scans for detecting AL. None of the on-site reports mentioned the existence of AL.
- Before a right thyroid lobectomy, patients usually do not benefit from a functional imaging modality combined with a CT scan. Since an RNRLN is more susceptible to damage, efforts should focus on the preoperative identification of AL 7 . US is the main preoperative imaging modality for assessing thyroid pathologies. It is also the cheapest and doesn’t require radiation exposure.

US is a simple, non-invasive, and effective method to easily detect the common Y-shaped division of the BCA. A systematic preoperative neck and chest CT scan 13 before thyroid surgeries 17 or thyroid cancer surgeries 25 can accurately identify AL but entails high healthcare costs and radiation exposure in order to identify a rare anatomical variant. An existing AL is rarely described in CT scan reports 3 17 25 and is considered inconsequential since its association with RNRLN is poorly known 26 . Considering our results, CT scan or MRI examination should only be used in specifically difficult US cases.

A previous review 11 listed 4 factors to avoid iatrogenic nerve injury, the first one being knowledge of potential anatomical variations, the other 3 being visual identification of at-risk nerves, intraoperative nerve monitoring, and the surgeon’s expertise. An RNRLN indeed increases intraoperative nerve damage risks 5 and subsequent vocal fold paralysis (12% vs. 1.8%) 10 . AL and its association with an RNRLN are poorly known 26 and should be taught to surgical residents 12 .

Visual RNRLN identification during surgery remains the reference standard for confirmation 27 . Surgical recommendations encourage systematic dissection of the RLN during thyroid and parathyroid surgeries 5 . Direct visualization of the RRLN/RNRLN is key to its preservation and has significantly reduced the global rate of RRLN palsy 28 . Failing to do so constitutes a major risk 27 .

Following standardized specific guidelines 9 , intraoperative neurophysiological monitoring (IONM) is recommended 16 29 , although not fully effective 12 , allowing a functional assessment of the RLN. It has been associated with a reduced risk of laryngeal paralysis with an RNRLN incidence of 2.7% to 6% 29 . Surgical direct visualization and IONM complement each other 5 7 8 . In cases where the nerve is not directly visualized and is not detected by IONM, being aware of the presence of an AL and its strong association with RNRLN is even more crucial 12 15 and is the surgeon’s “best defense” 5 . Some studies have leaned towards combining preoperative CT scan AL detection with IONM 16 17 25 . Our study establishes US as an accessible and effective method for complementary AL detection before surgery.

Detecting AL via imaging is easily done but is rarely mentioned by healthcare professionals 3 . AL and the RNRLN are distinct but strongly associated entities 16 17 . Only one isolated case of AL with a normal RRLN has ever been described 30 . Conversely, the presence of a Y-image does not exclude an RNRLN. Rare cases of RNRLNs can exist without AL 5 7 8 . Our study emphasizes how Y-image US detection suffices to exclude AL 14 . It is the first study to use the CT scan component of a PET/CT examination as the standard of truth for AL diagnosis. Previous studies 4 14 15 considered the absence of the Y-image on US to be positive for RNRLN when it should only suggest an existing AL 7 . Surgical false-positives for RNRLNs are possible 15 , corresponding to RRLN and cervical sympathetic trunk anastomoses. A left non-recurrent laryngeal nerve is an extremely rare variant 7 associated with conditions like a right aortic arch, dextrocardia, or situs inversus 5 23 .

Our study had several limitations. Firstly, it is a retrospective monocentric cohort. Our recruitment bias was due to an expected higher primary HPT prevalence in women, whereas renal HPT prevalence was the same for women and men. Secondly, all included patients underwent their low-dose CT scan on the same PET/CT machine, whereas the ultrasound examinations were performed by the same radiologist using various ultrasound equipment. This choice of a homogeneous dataset does not reflect day-to-day practice and does not account for inter-individual variations linked to ultrasound operators. We deliberately chose to limit our cohort to patients with US performed by the same expert radiologist who prospectively reported the presence or absence of the Y-image. Nevertheless, the assessment of the BCA division is simple and easy to check, even for juniors in our department. Our standardized US reports did not specify which probe was used (linear superficial or micro-convex/endo-cavitary probe) for the Y-image assessment. Huang et al. 14 reported a higher number of absent Y-images with a 10 MHz superficial probe (with 2 false-positives) compared to a 3.5 MHz probe. Thirdly, we also did not specify whether the direct origin of the RCCA from the aortic arch could be assessed. Combined with an RSCA postero-anterior trajectory (added value of US Doppler mode to characterize the artery flow), visualization of the direct origin of the RCCA from the aortic arch accounted for a PPV of 100%, and an overall accuracy of 98.9% in the 2015 study by Iacobone et al. 15 .

## 5. Conclusion

The detection of arteria lusoria (AL), which is strongly associated with a right non-recurrent laryngeal nerve (RNRLN), aims to reduce intraoperative nerve damage risks and subsequent vocal cord paralysis. Patients scheduled for thyroid/parathyroid surgery should undergo a systematic ultrasound examination of the neck and upper mediastinum to assess the presence or absence of the Y-image, reliably excluding AL with an NPV of 100%. Only when the Y-image is absent should low-dose CT be considered to confirm the supra-aortic anatomy and resolve false-positives.

## References

[1] Gunn A Oyekunle T Stang M Recurrent laryngeal nerve injury after thyroid surgery: An analysis of 11,370 patients J Surg Res 2020 255 42 49 10.1016/j.jss.2020.05.017 32540579

[2] Citton M Viel G Iacobone M Neck ultrasonography for detection of non-recurrent laryngeal nerve Gland Surg 2016 5 06 583 590 10.21037/gs.2016.11.07 28149804 PMC5233826

[3] Hermans R Dewandel P Debruyne F Arteria lusoria identified on preoperative CT and nonrecurrent inferior laryngeal nerve during thyroidectomy: A retrospective study Head Neck 2003 25 02 113 117 10.1002/hed.10180 12509793

[4] Yetisir F Salman AE Çiftçi B Efficacy of ultrasonography in identification of non-recurrent laryngeal nerve Int J Surg 2012 10 09 506 509 10.1016/j.ijsu.2012.07.006 22858799

[5] Henry BM Sanna S Graves MJ The non-recurrent laryngeal nerve: A meta-analysis and clinical considerations PeerJ 2017 5 e3012 10.7717/peerj.3012 28344898 PMC5363258

[6] Hanneman K Newman B Chan F Congenital variants and anomalies of the aortic arch Radiographics 2017 37 01 32 51 10.1148/rg.2017160033 27860551

[7] Bakalinis E Makris I Demesticha T Non-recurrent laryngeal nerve and concurrent vascular variants: A review Acta Med Acad 2018 47 02 186 196 10.5644/ama2006-124.230 30585070

[8] Kato K Toriumi Y Kamio M Nonrecurrent inferior laryngeal nerves and anatomical findings during thyroid surgery: Report of three cases Surg Case Rep 2016 2 44 10.1186/s40792-016-0170-5 PMC4870481 27188389

[9] Kim J Graves CE Jin C Intraoperative nerve monitoring is associated with a lower risk of recurrent laryngeal nerve injury: A national analysis of 17,610 patients Am J Surg 2021 221 02 472 477 10.1016/j.amjsurg.2020.10.013 33121660

[10] Toniato A Mazzarotto R Piotto A Identification of the nonrecurrent laryngeal nerve during thyroid surgery: 20-year experience World J Surg 2004 28 07 659 661 10.1007/s00268-004-7197-7 15175898

[11] Sharp E Roberts M Żurada-Zielińska A The most commonly injured nerves at surgery: A comprehensive review Clin Anat 2021 34 02 316 329 10.1002/ca.23696 33090551

[12] Zheng V Rajeev R Pinto D Variant anatomy of non-recurrent laryngeal nerve: When and how should it be taught in surgical residency? Langenbecks Arch Surg 2023 408 01 189 10.1007/s00423-023-02928-y 37160507

[13] Iacobone M Viel G Zanella S The usefulness of preoperative ultrasonographic identification of nonrecurrent inferior laryngeal nerve in neck surgery Langenbeck’s Arch Surg 2008 393 05 633 638 10.1007/s00423-008-0372-9 18604552

[14] Huang S Wu T Neck ultrasound for prediction of right nonrecurrent laryngeal nerve Head Neck 2010 32 07 844 849 10.1002/hed.21263 19953617

[15] Iacobone M Citton M Pagura G Increased and safer detection of nonrecurrent inferior laryngeal nerve after preoperative ultrasonography Laryngoscope 2015 125 07 1743 1747 10.1002/lary.25093 25960168

[16] Cai Q Guan Z Huang X The usefulness of preoperative computed tomography and intraoperative neuromonitoring identification of the nonrecurrent inferior laryngeal nerve Eur Arch Otorhinolaryngol 2013 270 07 2135 2140 10.1007/s00405-012-2336-5 23269396

[17] Gao E Zou X Zhou Y Increased prediction of right nonrecurrent laryngeal nerve in thyroid surgery using preoperative computed tomography with intraoperative neuromonitoring identification World J Surg Oncol 2014 12 262 10.1186/1477-7819-12-262 PMC4150955 25142438

[18] Polednak AP Anatomical variation in the right non-recurrent laryngeal nerve reported from studies using preoperative arterial imaging Surg Radiol Anat 2019 41 08 943 949 10.1007/s00276-019-02252-5 31087139

[19] Lee YS Son EJ Chang H Computed tomography is useful for preoperative identification of nonrecurrent laryngeal nerve in thyroid cancer patients Otolaryngol Head Neck Surg 2011 145 02 204 207 10.1177/0194599811406670 21521891

[20] Mediouni A Sayadi H Chahed H Non-recurrent laryngeal nerve and arteria lusoria: Rare and little-known association Clin Case Rep 2021 9 07 e04534 10.1002/ccr3.4534 PMC8385457 34466267

[21] Satoh S Tachibana S Yokoi T Preoperative diagnosis of nonrecurrent inferior laryngeal nerve Nippon Jibiinkoka Gakkai Kaiho 2013 116 07 793 799 10.3950/jibiinkoka.116.793 23980485

[22] Buffoli B Verzeletti V Hirtler L Retroesophageal right subclavian artery associated with a bicarotid trunk and an ectopic origin of vertebral arteries Surg Radiol Anat 2021 43 09 1491 1496 10.1007/s00276-021-02746-1 PMC8408090 33856505

[23] Henry JF Audiffret J Denizot A The nonrecurrent inferior laryngeal nerve: Review of 33 cases, including two on the left side Surgery 1988 104 06 977 984 3057672

[24] Petranović Ovčariček P Giovanella L Carrió Gasset I The EANM practice guidelines for parathyroid imaging Eur J Nucl Med Mol Imaging 2021 48 09 2801 2822 10.1007/s00259-021-05334-y PMC8263421 33839893

[25] Deveze A Sebag F Hubbard J Identification of patients with a non-recurrent inferior laryngeal nerve by duplex ultrasound of the brachiocephalic artery Surg Radiol Anat 2003 25 3/4 263 269 10.1007/s00276-003-0135-9 12819946

[26] Talbot JF Périé S Tassart M The presence of an arteria lusoria should be checked when reporting 18F-FCH PET/CT performed to localize hyperfunctioning parathyroid glands Clin Nucl Med 2023 48 11 958 959 10.1097/RLU.0000000000004814 PMC10581409 37756414

[27] Dralle H Sekulla C Haerting J Risk factors of paralysis and functional outcome after recurrent laryngeal nerve monitoring in thyroid surgery Surgery 2004 136 06 1310 1322 10.1016/j.surg.2004.07.018 15657592

[28] Hermann M Alk G Roka R Laryngeal recurrent nerve injury in surgery for benign thyroid diseases Ann Surg 2002 235 02 261 268 10.1097/00000658-200202000-00015 11807367 PMC1422423

[29] Donatini G Carnaille B Dionigi G Increased detection of non-recurrent inferior laryngeal nerve (NRLN) during thyroid surgery using systematic intraoperative neuromonitoring (IONM) World J Surg 2013 37 01 91 97 10.1007/s00268-012-1782-y 22955954

[30] Pelizzo MR Boschin IM Chondrogiannis S Aberrant right subclavian artery (“arteria lusoria”) without the known associated nerve anomaly: An “anomaly of the anomaly”? A clinical case and review of the literature Surg Radiol Anat 2017 39 08 927 931 10.1007/s00276-017-1831-1 28321495

